# Prognostic implications of predialysis patients’ symptoms in peritoneal dialysis patients

**DOI:** 10.1080/0886022X.2021.1871920

**Published:** 2021-01-21

**Authors:** Fang-Yu Wang, Zhuan Cui, Chun-yan Su, Tao Wang, Wen Tang

**Affiliations:** Department of Nephrology, Peking University Third Hospital, Beijing, China

**Keywords:** Peritoneal dialysis, symptom, mortality, outcomes

## Abstract

**Background:**

As kidney disease progresses, patients often experience a variety of symptoms. There are very few studies reporting spectrum of predialysis patients’ symptoms in peritoneal dialysis (PD) patients. Furthermore, the clinical significance of predialysis patients’ symptoms for PD patients’ prognosis remains unknown.

**Methods:**

In this retrospective cohort study, patients who started PD during 1 January 2006 to 31 January 2018 were included. Patients’ predialysis symptoms and clinical parameters were obtained. Both the short- and long-term patients’ outcome were investigated by Cox regression and Kaplan–Meier’s survival analysis to identify the relationship between clinical symptoms and patients' mortality on PD.

**Results:**

A total of 898 incident PD patients were included. The anorexia (58%) was the most common predialysis symptom in the present cohort, followed by insomnia (32.7%), fatigue (27.6%), syndromes of heart failure (27.6%), and nausea (20.5%). The only symptom significantly associated with both six-months and 12-months mortality on PD was nausea (HR 2.359, 95% CI 1.377–4.040, *p*=.002 and HR 1.791, 95% CI 1.176–2.729, *p*=.007, respectively). But in the long-term, anorexia (HR 1.392, 95% CI 1.070–1.811, *p*=.014) was the only symptom significantly associated with patient's all-cause mortality after adjusting for other confounding factors.

**Conclusions:**

Our study demonstrated that nausea and anorexia were the most important predialysis symptoms, which was associated with patients’ short- and long-term mortality on PD treatment, respectively. The results indicated that predialysis evaluation and management of symptoms of nausea and anorexia may be a possible way to improve patients’ outcome on PD.

## Background

As kidney disease progresses, patients often experience a variety of symptoms [1,2], such as anorexia, fatigue, cognitive impairment, depressive symptoms, pruritus, and sleep disturbances [3]. As showed by the IDEAL study [4] that 76% of the patients allotted to late dialysis had to start dialysis before the target of estimated glomerular filtration rate (eGFR) <7 ml/min due to uremic symptoms. These further supported the notion that the predialysis symptoms are crucial in clinic setting for clinician to decide the right timing to begin dialysis [4,5].

To identify the most meaningful symptoms and accordingly, to decide the right timing of dialysis initiation is still a challenge for the nephrologists. However, there are very few studies reporting spectrum of predialysis patients’ symptoms in peritoneal dialysis (PD) patients and little is known about the burden of symptoms of predialysis patients especially PD patients [6]. In addition, unlike many predialysis indictors linking to the patients outcomes, such as GFR, serum albumin [7,8], calcium [9,10], body mass index [11], and comorbidities [8,12], the potential predictive value of patients’ predialysis symptoms have not been fully elucidated.

In the present study, we investigated spectrum of predialysis symptoms and tried to identify the most important predialysis symptoms linking to patients’ short-term and long-term outcomes.

## Methods

In this retrospective cohort study, all incident patients who started PD therapy from 1 January 2006 to 31 January 2018 from Peking University Third Hospital were included. Patients were excluded if they had received any form of dialysis for more than 1 month, or if they had received previous kidney transplantation. Patients who started PD in other center were also excluded for we were unable to get their detailed predialysis symptom information. All the patients were followed by PD clinic of Peking University Third Hospital. Patients were followed until death, cessation of PD or end of study as of 15 November 2018. Patients’ medical charts were reviewed to extract the information. In our center, predialysis patients need to be hospitalized to department of nephrology for performing PD catheter implantation. Therefore, patients’ detailed predialysis clinic symptoms, predialysis manifestations, clinical history, demographics, lab assay were recorded when admitted. During the study period, patients’ hospitalization medical charts were carefully reviewed to obtain the predialysis symptom data.

The study protocol was approved by the Peking University Third Hospital Medical Science Research Ethics Committee, IRB00006761-M2019290. Review of patients’ records and use of data by this study were permitted by Peking University Third Hospital Medical Science Research Ethics Committee. Patients’ eGFR was estimated by Chronic Kidney Disease Epidemiology Collaboration (CKD-EPI) creatinine equation [13]. Hypocalcemia were defined as fasting plasma calcium level lower than 2.0 mmol/l and hyperphosphatemia were defined as fasting plasma phosphorus level higher than 1.78 mmol/l.

The primary outcomes examined were all-cause mortality on PD (censored for loss to follow-up, renal transplantation, recovery of renal function, transferred to hemodialysis (HD) due to technical failure, transfer to other dialysis centers and end of study).

### Statistical analysis

Results were expressed as frequencies and percentages for categorical variables, mean ± standard deviation for continuous normally distributed variables, and median (interquartile range, IQR) for continuous variables that were not normally distributed. Comparison for patients in different groups was performed using chi-square tests, two-tailed unpaired *t*-tests or Mann–Whitney’s tests, depending on data distribution. Mortality risks (both 0.5, 1 year and long-term mortality) were analyzed by the Kaplan–Meier and multivariate Cox’s proportional hazard model in which all the significant variables (*p*<.1) from the univariate analysis were included. Statistical analysis was performed using IBM SPSS software, version 22.0 (Armonk, NY). *p* Values less than .05 were considered statistically significant.

## Results

**Figure 1.**
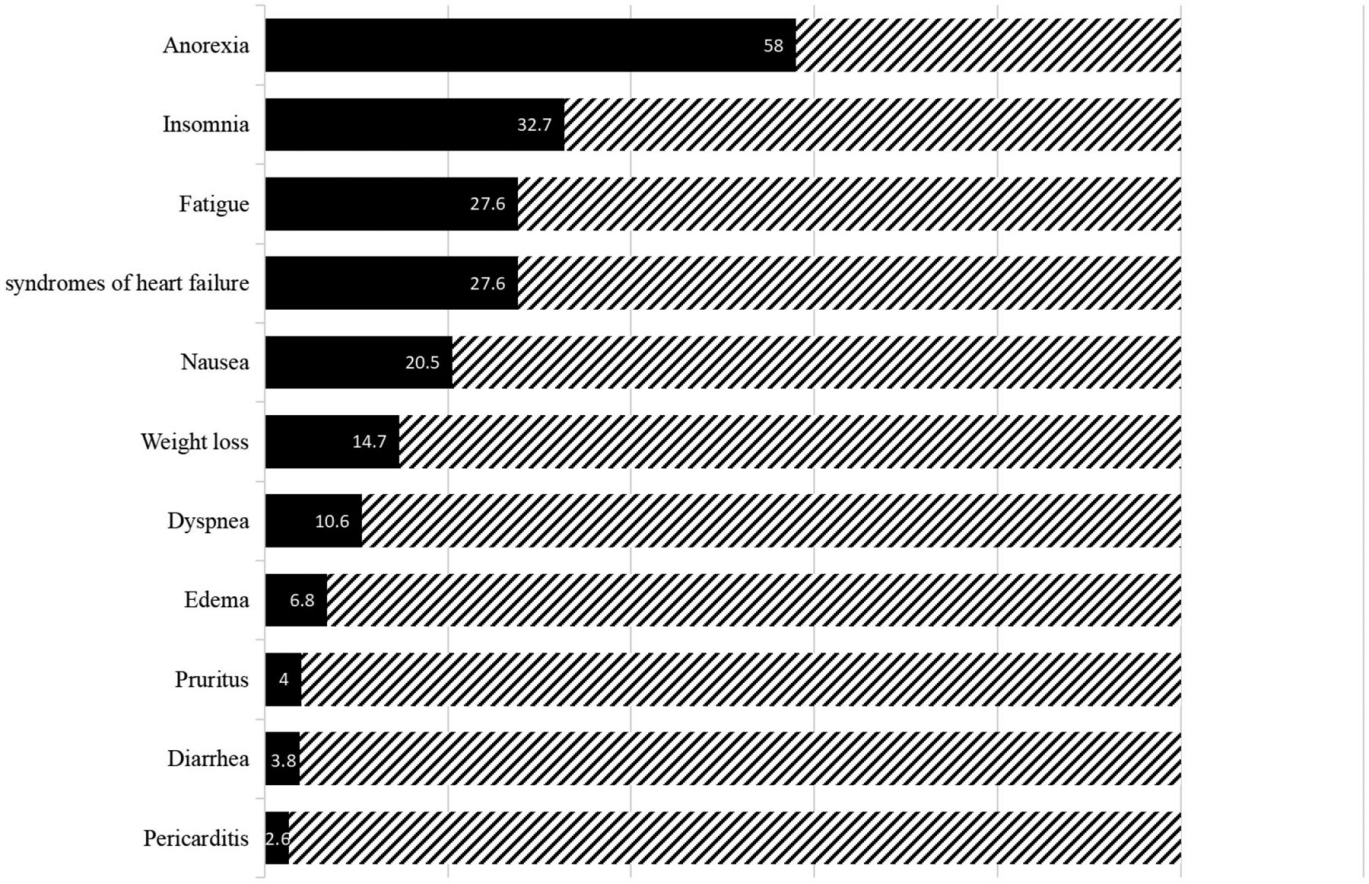
Patients’ uremic manifestations and syndromes at the initiation of peritoneal dialysis in 898 patients.

Clinical and laboratory characteristics at the initiation of PD are depicted in Table 1. Patients’ uremic manifestations and syndromes at the initiation of PD are shown in Figure 1. The anorexia (58%) was the most common symptom in the present cohort, followed by insomnia (32.7%), fatigue (27.6%), syndromes of heart failure (27.6%), and nausea (20.5%), respectively.

**Table 1.** Clinical and laboratory characteristics at the time of peritoneal dialysis initiation in 898 patients with CKD.

	Mean ± SD	Median (IQ range)
Patients characteristics		
Age (years)	59.55 ± 16.34	61.31 [48.98–72.49]
Males (%)	50.9	
Height (cm)	163.6 ± 8.4	163.0 [157.0–170.0]
Weight (kg)	62.8 ± 12.7	62.0 [54.0–71.0]
BMI (kg/m^2^)	23.4 ± 3.8	23.1 [20.8–25.6]
Laboratory parameters		
Creatinine (μmol/l)	803 ± 345	734 [570–966]
Urea (mmol/l)	29.5 ± 10.5	28.9 [22.3–35.3]
Albumin (g/l)	35.5 ± 5.4	36.0 [31.8–39.6]
Hemoglobin (g/l)	85.1 ± 19.2	84.0 [72.0–98.0]
Calcium (mmol/l)	1.96 ± 0.31	1.98 [1.80–2.14]
Phosphate (mmol/l)	1.94 ± 0.59	1.98 [1.51–2.31]
Parathyroid hormone (pg/ml)	307 ± 259	245 [138–417]
Urine volume (ml)	1065 ± 583	1000 [650–1401]
eGFR (ml/min.1.73 m^2^)	6.08 ± 3.13	5.33 [4.02–7.42]
Comorbidities	Percent	
Acute heart failure	27.6	
Pulmonary infection	12.5	
Diabetes mellitus	38.3	
Coronary heart disease	19.3	
Old myocardial infarction	5.8	
Cerebral infarction	11.9	
Cerebral hemorrhage	2.4	
Amputation	0.4	
Uremia encephalopathy	0.7	
Hyperkalemia	7.5	
Acidosis	52.2	
Hypocalcemia	48.7	
Hyperphosphatemia	48.3	
Hypertension	94	

CKD: chronic kidney disease; CI: confidence interval; BMI: body mass index; eGFR: estimated glomerular filtration rate.

Acidosis is defined as CO2CP <23 mmol/l; hyperkalemia is defined as potassium >5.5 mmol/l; hypocalcemia is defined as serum calcium level <2.00; hyperphosphatemia is defined as serum phosphorus >1.78 mmol/l.

All the patients were followed until 15 November 2018. A total of 898 incident PD patients with a median follow-up of 3.73 (inter-quarter range 1.13, 5.56) years were included in the present study. Death occurred in 384 (42.8%) patients. The causes of death were cardiovascular disease (38%), peritonitis (6.5%), multiple organ failure (9.6%), infection (14.1%), tumor (6.8%), gastrointestinal bleeding (6.5%), respiratory failure (6%), withdraw of therapy (4.4%), and unknown reasons (8.1%).

A total of 28 patients (3.1%) transferred to HD. Fifty-one patients (5.6%) underwent renal transplantation and five patients (0.5%) had recovery of renal function during follow-up. Loss to follow-up occurred in six patients (0.6%) and 20 patients (2.2%) transferred to other dialysis centers.

### Patients’ symptoms and short-term outcome on PD

Both the six-months and 12-month mortality were evaluated in the present study for patients’ short-term outcome. Univariate Cox regression analysis showed that the only symptom significantly associated with both six-months and 12-months mortality on PD was nausea (HR 2.359, 95% CI 1.377–4.040, *p*=.002 and HR 1.791, 95% CI 1.176–2.729, *p*=.007, respectively) (Table 2).

**Table 2.** Cox regression model with predialysis patients’ symptoms included to investigate predictors for short-term mortality for patients underwent peritoneal dialysis.

	Six months mortality				One year mortality			
	Univariate Cox regression		Multivariate Cox regression		Univariate Cox regression		Multivariate Cox regression	
Variable	HR [95% CI]	*p*	HR [95% CI]	*p*	HR [95% CI]	*p*	HR [95% CI]	*p*
Patients characteristics								
Age (per 1-year increase)	1.046 [1.025–1.067]	<.001	1.070 [1.034–1.107]	<.001	1.051 [1.035–1.067]	<.001	1.055 [1.032–1.079]	<.001
Gender (male)	1.430 [0.845–2.419]	.183			1.288 [0.873–1.900]	.201	0.840 [0.507–1.391]	.497
BMI (kg/m^2^)	0.884 [0.816–0.958]	.002	0.939 [0.845–1.044]	.247	0.912 [0.862–0.965]	.001	0.953 [0.884–1.027]	.208
Laboratory parameters								
Creatinine (per 1-μmol/l increase)	0.999 [0.998–1.000]	.035	1.000 [0.999–1.001]	.955	0.999 [0.998–0.999]	<.001		
Urea (per 1-mmol/l increase)	0.987 [0.962–1.013]	.317			0.965 [0.945–0.985]	.001	0.982 [0.951–1.014]	.277
Albumin (per 1-g/l increase)	0.876 [0.835–0.919]	<.001	0.878 [0.814–0.947]	.001	0.890 [0.859–0.922]	<.001	0.902 [0.856–0.950]	<.001
Hemoglobin (per 1-g/l increase)	1.006 [0.993–1.020]	.354			1.006 [0.996–1.016]	.240		
Calcium (per 1-mmol/l increase)	2.176 [0.943–5.019]	.068	2.386 [0.649–8.780]	.191	2.192 [1.176–4.083]	.013	1.749 [0.712–4.296]	.223
Phosphorus (per 1-mmol/l increase)	0.612 [0.375–0.999]	.049	1.168 [0.602–2.266]	.646	0.581 [0.403–0.838]	.004	1.166 [0.712–1.910]	.541
Parathyroid hormone (per 1-pg/ml increase)	0.998 [0.996–0.999]	.011	1.000 [0.998–1.002]	.981	0.998 [0.997–0.999]	.002	1.000 [0.999–1.001]	.829
Urine volume (ml)	0.999 [0.998–0.999]	<.001	0.999 [0.998–1.000]	.044	0.999 [0.998–0.999]	<.001	0.999 [0.998–1.000]	<.001
eGFR (per 1-ml/min.1.73 m^2^ increase)	1.045 [0.975–1.120]	.217			1.057 [1.003–1.113]	.038	0.946 [0.858–1.042]	.256
Hyperkalemia	0.933 [0.338–2.579]	.894			0.772 [0.338–1.760]	.538		
Acidosis	1.168 [0.692–1.970]	.561			0.971 [0.659–1.428]	.879		
Comorbidities								
Acute heart failure	1.215 [0.695–2.124]	.494			1.350 [0.898–2.029]	.149		
Pulmonary infection	1.199 [0.568–2.531]	.634			1.890 [1.170–3.052]	.009	1.507 [0.826–2.749]	.181
Diabetes mellitus	1.153 [0.681–1.950]	.596			1.510 [1.026–2.222]	.037	1.350 [0.802–2.273]	.259
Coronary heart disease	1.469 [0.814–2.648]	.201			1.320 [0.841–2.071]	.227		
Old myocardial infarction	1.987 [0.853–4.629]	.112			1.852 [0.964–3.555]	.064	1.540 [0.682–3.479]	.299
Cerebral infarction	1.582 [0.800–3.131]	.188			1.488 [0.884–2.503]	.134		
Cerebral hemorrhage	2.186 [0.684–6.992]	.187			2.529 [1.109–5.769]	.027	5.303 [1.813–15.508]	.002
Predialysis symptoms								
Dyspnea	1.564 [0.768–3.188]	.218			1.447 [0.837–2.502]	.186		
Hypertension	0.282 [0.143–0.559]	<.001	0.776 [0.229–2.631]	.684	0.546 [0.284–1.048]	.069	1.283 [0.388–4.245]	.683
Pericarditis	2.149 [0.672–6.874]	.197			1.983 [0.807–4.871]	.135		
Edema	1.006 [0.364–2.780]	.991			1.431 [0.746–2.748]	.281		
Fatigue	1.456 [0.845–2.509]	.176			1.329 [0.881–2.003]	.175		
Anorexia	1.254 [0.732–2.147]	.410			1.307 [0.874–1.995]	.192		
Nausea	2.359 [1.377–4.040]	.002	2.282 [1.085–4.797]	.030	1.791 [1.176–2.729]	.007	2.004 [1.180–3.402]	.010
Weight loss	1.102 [0.541–2.245]	.790			0.846 [0.473–1.513]	.573		
Pruritus	0.419 [0.058–3.026]	.388			1.480 [0.649–3.375]	.352		
Insomnia	0.669 [0.366–1.222]	.191			1.010 [0.670–1.522]	.963		
Diarrhea	1.422 [0.444–4.547]	.553			1.683 [0.738–3.840]	.216		

CI: confidence interval; BMI: body mass index; eGFR: estimated glomerular filtration rate.

Multivariate Cox’s regression model included all the significant variables (*p*<.1) from the univariate analysis. Acidosis is defined as CO2CP <23 mmol/l; hyperkalemia is defined as potassium >5.5 mmol/l.

**Figure 2.**
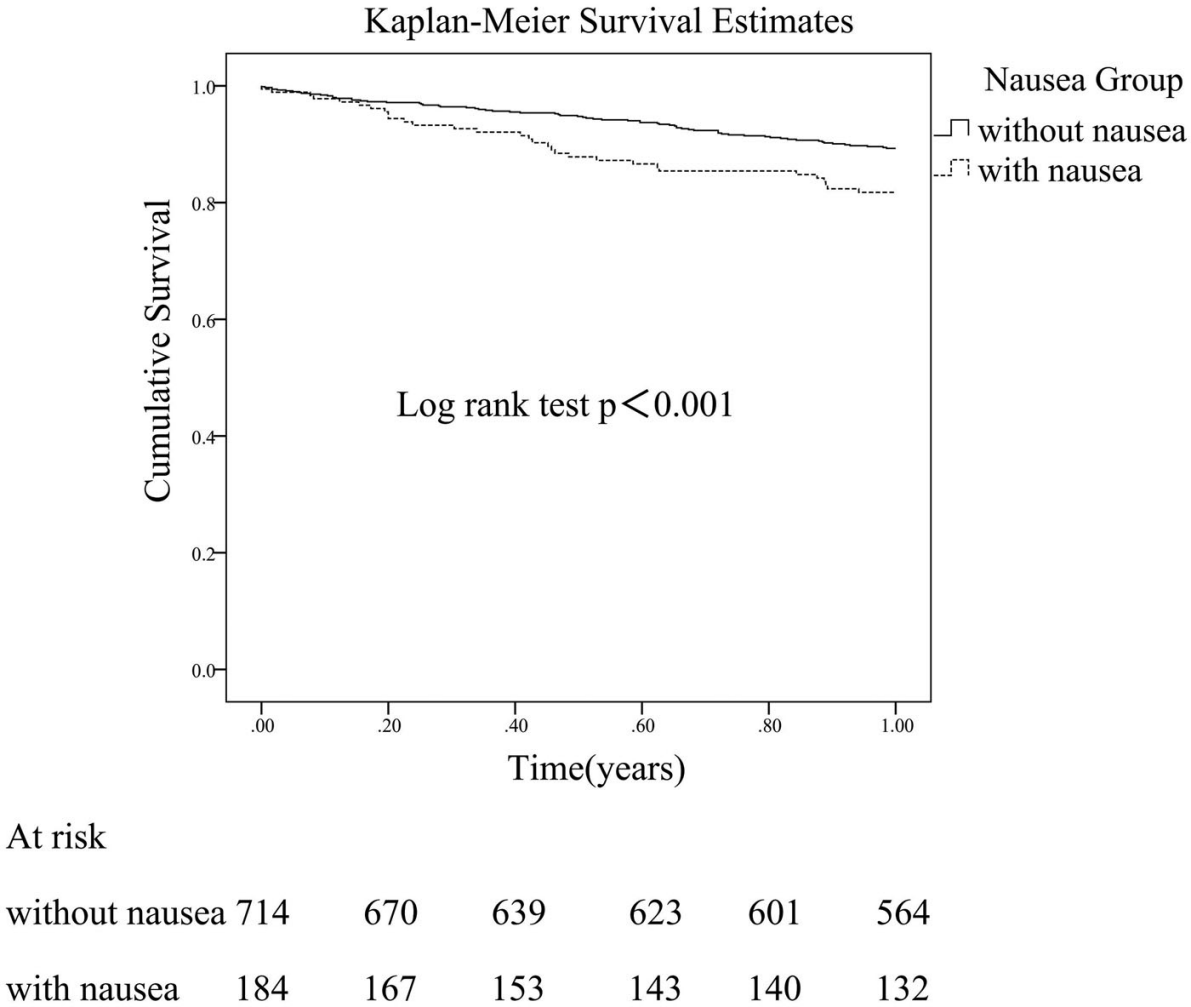
Kaplan–Meier’s one-year survival curve for peritoneal dialysis patients with or without predialysis symptom of nausea.

Both the six-months and 12-month survival time of patients with symptom of nausea were significantly lower compared to patients without (log rank test, both *p*<.001) (Figure 2). Multivariate Cox regression analysis showed symptoms of nausea (HR 2.282, 95% CI 1.085–4.797, *p*=.030) was significantly associated with patient’s all-cause mortality in six month after adjusting for age (HR 1.070, 95% CI 1.034–1.107, *p*<.001), serum albumin (HR 0.878, 95% CI 0.814–0.947, *p*=.001), urine volume (HR 0.999, 95% CI 0.998–1.000, *p*=.044), and other confounding factors. Similarly, multivariate Cox regression analysis showed symptoms of nausea (HR 2.004, 95% CI 1.180–3.402, *p*=.010) was significantly associated with patient’s all-cause mortality in 12 months after adjusting for age (HR 1.055, 95% CI 1.032–1.079, *p*<.001), serum albumin (HR 0.902, 95% CI 0.856–0.950, *p*<.001), urine volume (HR 0.999, 95% CI 0.998–1.000, *p*<.001), with cerebral hemorrhage (HR 5.303, 95% CI 1.813–15.508, *p*=.002) and other confounding factors.

### Patients’ symptoms and long-term mortality on PD

**Figure 3.**
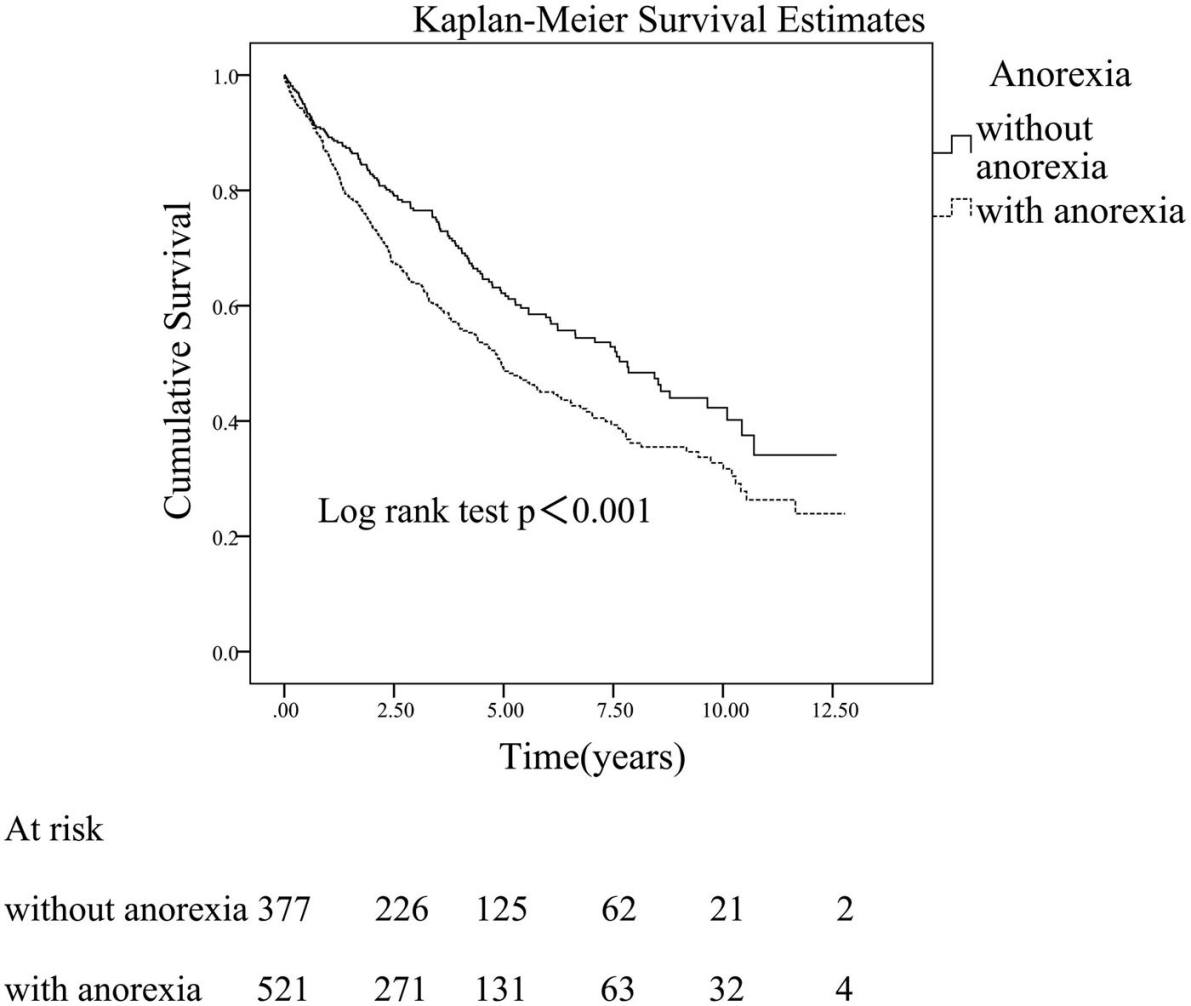
Kaplan–Meier’s survival curve for peritoneal dialysis patients with or without predialysis symptom of anorexia.

Univariate Cox regression analysis showed that the predialysis symptoms significantly associated with patients’ all-cause mortality were dyspnea (*p*=.001), edema (*p*=.016), nausea (*p*=.033), anorexia (*p*<.001), and insomnia (*p*=.008) (Table 3). Interestingly, multivariate Cox regression analysis showed that anorexia (HR 1.392, 95% CI 1.070–1.811, *p*=.014) was the only symptom significantly associated with patients’ all-cause mortality after adjusting for other confounding factors (Table 3). The survival time of patients with symptom of anorexia was significantly lower compared to patients who without anorexia (log rank test, *p*<.001) (Figure 3).

**Table 3.** Cox regression model with predialysis patients’ symptoms included to investigate predictors for long-term mortality for patients underwent peritoneal dialysis.

	Univariate Cox regression		Multivariate Cox regression	
Variable	HR [95% CI]	*p*	HR [95% CI]	*p*
Patient characteristics				
Age (per 1-year increase)	1.050 [1.042–1.059]	<.001	1.051 [1.040–1.063]	<.001
Gender (male)	0.987 [0.808–1.206]	.901		
BMI (kg/m^2^)	0.958 [0.932–0.985]	.003	0.984 [0.952–1.018]	.354
Laboratory parameters				
Creatinine (per 1-μmol/l increase)	0.999 [0.998–0.999]	<.001	1.000 [0.999–1.001]	.629
Urea (per 1-mmol/l increase)	0.979 [0.969–0.989]	<.001	1.004 [0.988–1.020]	.602
Albumin (per 1-g/l increase)	0.944 [0.926–0.962]	<.001	0.951 [0.927–0.976]	<.001
Hemoglobin (per 1-g/l increase)	1.005 [1.000–1.010]	.064	1.000 [0.993–1.006]	.975
Calcium (per 1-mmol/l increase)	1.963 [1.445–2.667]	<.001	2.647 [1.713–4.092]	<.001
Phosphorus (per 1-mmol/l increase)	0.655 [0.542–0.791]	<.001	1.024 [0.783–1.338]	.864
Parathyroid hormone (per 1-pg/ml increase)	0.999 [0.998–0.999]	<.001	1.000 [0.999–1.001]	.879
Urine volume (ml)	0.999 [0.999–1.000]	<.001	1.000 [0.999–1.000]	.002
eGFR (per 1-ml/min.1.73 m^2^ increase)	1.077 [1.048–1.107]	<.001	1.005 [0.942–1.072]	.884
Hyperkalemia	1.231 [0.861–1.759]	.254		
Acidosis	0.889 [0.727–1.087]	.251		
Comorbidities				
Acute heart failure	1.352 [1.095–1.669]	.005	1.226 [0.919–1.635]	.166
Pulmonary infection	1.448 [1.098–1.908]	.009	1.097 [0.788–1.527]	.583
Diabetes mellitus	1.725 [1.410–2.110]	<.001	1.344 [1.041–1.736]	.023
Coronary heart disease	1.772 [1.414–2.219]	<.001	0.925 [0.672–1.274]	.634
Old myocardial infarction	2.030 [1.419–2.904]	<.001	1.247 [0.773–2.013]	.366
Cerebral infarction	1.388 [1.042–1.848]	.025		
Cerebral hemorrhage	1.458 [0.838–2.536]	.182		
Predialysis symptoms				
Dyspnea	1.664 [1.238–2.236]	.001	1.241 [0.844–1.827]	.273
Hypertension	0.734 [0.502–1.072]	.11		
Pericarditis	1.013 [0.541–1.899]	.967		
Edema	1.507 [1.078–2.106]	.016	1.083 [0.710–1.651]	.713
Fatigue	1.190 [0.958–1.478]	.116		
Anorexia	1.455 [1.181–1.792]	<.001	1.392 [1.070–1.811]	.014
Nausea	1.289 [1.021–1.627]	.033	1.204 [0.905–1.602]	.202
Weight loss	0.913 [0.677–1.233]	.554		
Pruritus	1.523 [0.948–2.446]	.082	1.186 [0.678–2.076]	.550
Insomnia	1.328 [1.078–1.637]	.008	0.999 [0.771–1.295]	.995
Diarrhea	1.115 [0.665–1.869]	.681		

CI: confidence interval; BMI: body mass index; eGFR: estimated glomerular filtration rate.

Multivariate Cox’s regression model included all the significant variables (*p*<.1) from the univariate analysis. Acidosis is defined as CO2CP <23 mmol/l; hyperkalemia is defined as potassium >5.5 mmol/l.

## Discussion

In this retrospective cohort study, we found that the most common predialysis symptom was anorexia. Most importantly, we demonstrated that predialysis anorexia was a strong and significant predictor for PD patients’ long-term mortality. In addition, presenting with symptom of nausea was associated with high short-term PD mortality after adjusting for all the other confounding factors.

Anorexia (58%) was the most common symptom in the present cohort, followed by insomnia (32.7%), fatigue (27.6%), syndromes of heart failure (27.6%), and nausea (20.5%). These results are different from the previous study that the most common symptoms were fatigue (44%), nausea (24%), and anorexia (22%) at the dialysis initiation [6]. Nausea and anorexia are major uremic symptoms and a frequent indication for starting dialysis [14]. The higher present of anorexia and nauseas may reflect a relatively late start of dialysis in the present predialysis patients. Interestingly, we found that nausea is a more powerful predictor for patients’ short-term (6 and 12 months) mortality than anorexia after adjusting other confounding factors, which indicated that for patients with symptoms of nausea, more intensive treatment may be needed to ameliorate the symptom and thus to improve patients short-term outcome.

Previous study in PD patients showed that self-reported appetite (anorexia) was a predictor of clinical characteristics and outcome for patients receiving PD [15] and HD patients [16]. Different from this study which anorexia were evaluated on PD at least 12 weeks or on HD for at least 120 days [16], symptom of anorexia was evaluated just before PD initiation in the present study. The symptoms of anorexia in the present study reflect predialysis patients’ status rather than after dialysis, which may indicate that in advance chronic kidney disease (CKD), the symptoms of anorexia could be an indicator of long-term PD outcomes in spite that standard dialysis prescription was delivered. This result is consistent with the study from Thiane et al. who showed that in CKD5 non-dialysis patients, there was a significant increase in mortality risk in PD patients with poor appetite [16]. The underlining causes for anorexia could be accumulation of unidentified anorexigenic compounds, inflammatory cytokines, and alterations in appetite regulation [17,18]. This further contributes to the high prevalence of protein-energy wasting (PEW) and could have a direct adverse effect on morbidity and mortality of these patients [13].

Our study suggest that a single evaluation of predialysis symptoms of anorexia can help identify patients with high risk after PD, which suggested performing appetite evaluation periodically is crucial for patients with advance CKD and may help to decide the right timing for commencing maintenance dialysis treatment. Therefore, more intensive intervention and management either before or after PD treatment would be necessary for these patients. Further study is needed to elucidate the possible effect of appetite improvement by PD as well as its effect on clinical outcomes.

Our study has some limitations. First, patients included in this study were recruited from a single tertiary academic hospital in China, thereby raising the possibility of ascertainment bias. Second, due to the retrospective design, some detailed symptoms severity could not be obtained. Finally, although we attempted to adjust for a range of demographic, clinical, and laboratory characteristics, some detailed comorbidity index (e.g., Charlson comorbidity index and nutritional status assessment like subjective global nutrition assessment, SGA) was not collected, thus residual confounding remains possible.

## Conclusions

Our study demonstrated that predialysis nausea and anorexia were the most important predialysis symptoms, which was associated with patients’ short-term and long-term mortality on PD treatment, respectively. These results indicated that predialysis evaluation and management of symptoms of nausea and anorexia may be a possible way to improve patients’ outcome on PD.

## Data Availability

The datasets used during the current study are available from the corresponding author on reasonable request.

## Abbreviations

PD: peritoneal dialysis
CKD: chronic kidney disease
eGFR: estimated glomerular filtration rate
CKD-EPI: Chronic Kidney Disease Epidemiology Collaboration creatinine equation
IQR: interquartile range
HD: hemodialysis
PEW: protein-energy wasting
